# O-GLcNAc Post-Translational Modifications Regulate the Entry of Neurons Into an Axon Branching Program

**DOI:** 10.1002/dneu.20695

**Published:** 2008-12-11

**Authors:** Herb Francisco, Katherine Kollins, Neal Varghis, David Vocadlo, Keith Vosseller, Gianluca Gallo

**Affiliations:** 1 Department of Neurobiology, Drexel University College of MedicinePhiladelphia, Pennsylvania 19129; 2 Department of Biochemistry, Drexel University College of MedicinePhiladelphia, Pennsylvania 19102; 3 Department of Chemistry, Simon Fraser UniversityBurnaby, British Columbia, Canada V5A 1S6

**Keywords:** O-GlcNAc, growth cone, axon, polarity, filopodium, filopodia, cAMP, PKA

## Abstract

Many neuronal cytosolic and nuclear proteins are post-translationally modified by the reversible addition of O-linked *N*-acetylglucosamine (O-GlcNAc) on serines and threonines. The cellular functions of O-GlcNAc modifications in neuronal development are not known. We report that O-GlcNAc-modified proteins are distributed nonuniformly throughout cultured primary chicken forebrain neurons, with intense immunostaining of the cell body, punctuate immunostaining in axons and all processes, and localization in filopodia/lamellipodia. Overexpression of O-GlcNAcase, the enzyme that removes O-GlcNAc from proteins, increased the percentage of neurons exhibiting axon branching without altering the frequency of axon branches on a per neuron basis and increased the numbers of axonal filopodia. Conversely, pharmacologically increasing O-GlcNAc levels on proteins through specific inhibition of O-GlcNAcase with the inhibitor 9d decreased the numbers of axonal filopodia, but had no effect on axon length or branching. Treatment with an alternative O-GlcNAcase inhibitor, PUGNAc, similarly decreased the number of axonal filopodia. Furthermore, axon branching induced by the adenylyl cyclase activator forskolin was suppressed by pharmacological inhibition of O-GlcNAcase. Western analysis revealed that O-GlcNAc levels regulate the phosphorylation of some PKA substrates in response to forskolin. These data provide the first evidence of O-GlcNAc modification-specific influences in neuronal development in primary culture, and indicate specific roles for O-GlcNAc in the regulation of axon morphology. © 2008 Wiley Periodicals, Inc. Develop Neurobiol 69: 162–173, 2009

## INTRODUCTION

Post-translational modification of proteins is a fundamental mechanism for the regulation of cellular functions. In contrast to luminal and extracellular complex glycosylation, O-linked *N*-acetylglucos-amine (O-GlcNAc) is a single *N*-acetylglucsosamine moiety linked to serines and threonines of cytosolic and nuclear proteins (Hart, 1997). O-GlcNAc is dynamic in response to cell stimuli, and appears to act as a widespread regulatory modification in many ways analogous to phosphorylation (Slawson et al., 2006). In the same way the kinases and phosphatases dynamically regulate phosphorylation, cytosolic and nuclear enzymes catalyze addition (OGT) and removal (O-GlcNAcase) of O-GlcNAc (Kreppel et al., 1997; Gao et al., 2001). O-GlcNAc is found in all multicellular eukaryotes and has been implicated in a variety of fundamental cellular processes such as signal transduction (Wells et al., 2001). Mechanistic examples of site-specific O-GlcNAc regulatory function are limited, but include influences on protein–protein interactions, subcellular localization, protein half-life, and interplay with phosphorylation (Kamemura and Hart, 2003). O-GlcNAc and the enzymes that add and remove O-GlcNAc are most abundant in brain (Gao et al., 2001; Okuyama and Marshall, 2003), and OGT is enriched at neuronal synapses (Cole and Hart, 2001) where it localizes to synaptic vesicles (Akimoto et al., 2003). Consistent with these observations, an abundance of O-GlcNAc is observed on proteins known to function in neuronal vesicular transport and recycling (e.g., synapsin I, bassoon, and piccolo; Vosseller et al., 2006). O-GlcNAc is also abundant on proteins involved in the regulation of the microtubule (Ding and Vandré, 1996; Liu et al., 2004) and neurofilament (Dong et al., 1993) cytoskeleton. Changes in O-GlcNAc levels on neuronal proteins have been correlated with neurological disease states such as Alzheimer's disease (Griffith and Schmitz, 1995; Lefebvre et al., 2005). Neuron-specific knock out of OGT results in motor impairments and early neonatal lethality (O'Donnell et al., 2004), suggesting that the lack of O-GlcNAc transferase activity impairs nervous system development. Although critical evolutionarily conserved functions for O-GlcNAc on neuronal proteins have been suggested, the roles of O-GlcNAc in living neurons are not clear.

O-GlcNAc regulatory function may occur through competition with phosphorylation on specific serines/threonines residues of target proteins (reviewed in Zachara and Hart, 2006). Indeed, functional reciprocity between O-GlcNAc and phosphorylation on Tau appears to contribute to neurofibrillary tangle formation in Alzheimer's disease (Liu et al., 2004). Phosphorylation is a critical mechanism in axon development, and O-GlcNAc has been implicated in the regulation of signaling through kinases in neurons and glia (e.g., PKA, PKC; Griffith and Schmitz, 1999; Matthews et al., 2005). Thus, O-GlcNAc may regulate aspects of neuronal development in concert with phosphorylation.

We investigated the distribution of O-GlcNAc-modified proteins in cultured forebrain neurons and determined the effects of experimentally altering levels of O-GlcNAc on primary neuronal development. Our results indicate that O-GlcNAc modification of proteins in developing primary neurons has specific functions in the regulation of axonal filopodial and branch formation.

## MATERIALS AND METHODS

### Culturing and Pharmacological Reagents

Chicken embryonic Day 8 forebrain neurons were prepared following the protocol of Heidemann et al. (2003). Briefly, neurons were cultured in 500 μL of M199 medium containing 10% fetal bovine serum and N9 supplements at a density of 5 × 10^4^ neurons per 18 × 18 mm German glass coverslip. Coverslips were coated overnight with 100 μg/mL polylysine (Sigma, St. Louis, MO) in borate buffer.

PUGNAc was from Carbogen (Bubendorf, Switzerland), 9d was synthesized and characterized in the laboratory of one of the authors (Vocadlo) as previously described (Macauley et al., 2005). Forskolin and Blebbistatin were purchased from Calbiochem (San Diego, CA) and Toronto Research Chemicals (North York, Canada), respectively.

### Transfection

Transfection was performed using the Amaxa Nucleofector reagents following the manufacturer's directions (program 0-003, 10^6^ cells; Amaxa, Cologne, Germany). Experimental neurons were cotransfected with 7 μg of rat O-GlcNAcase cloned in the pcDNA3.1 vector and 3 μg DsRed plasmid, control neurons were transfected with 3 μg DsRed plasmid alone (Clontech, Palo Alto, CA). Immediately following transfection, the neurons were resuspended in culturing medium and plated at 5 × 10^4^ cells per coverslip. The recombinant human O-GlcNAcase vector is a kind gift of Dr. Lance Wells. Generation of the construct is described in Wells et al. (2002).

### Immunocytochemistry

Neurons were fixed by a 15-min treatment with 0.25% glutaraldehyde and 0.1% Triton X-100 in PHEM buffer, followed by 15 min in 2 mg/mL sodium borohydride (Sigma), washed using phosphate buffered saline (PBS), blocked and extracted with a 15-min treatment of 10% goat serum in PBS containing 0.1% Triton X-100. This fixation allows for soluble cytoskeletal proteins to be extracted, while cytoskeletal polymers are fixed providing unambiguous detection of microtubules (Gallo and Letourneau, 1999). For morphometric analysis, microtubules were stained using a FITC-conjugated monoclonal anti tubulin primary antibody (DM1A, Sigma; 1:100, 45 min). Actin filaments were stained for 45 min using rhodamine phalloidin (Invitrogen, Carlsbad, CA) following manufacturer's directions. O-GlcNAc staining was determined using a mouse monoclonal anti-O-GlcNAc antibody (CTD 110.6; Comer et al., 2001; 1:250, 45 min followed by washing with PBS and treatment with a secondary antibody conjugated to FITC). All samples were mounted in anti fade medium to prevent bleaching.

### Quantification of O-GlcNAc Staining Intensity

Neurons were transfected with either dsRED or dsRED + O-GlcNAcase and cultured for 48 h, followed by fixation and staining with anti-O-GlcNAc antibodies as described above. Both groups were transfected, cultured, and stained in parallel. A set of dsRED-transfected cultures was stained with secondary antibody alone in order to determine background levels of staining. Images of the O-GlcNAc staining intensities in neuronal cell bodies were acquired using a 20× objective. Camera acquisition parameters were determined such that no image contained pixels at saturation and that cells in cultures stained with only secondary antibody only minimally registered, thereby establishing the dynamic range for measurement from background fluorescence to maximal staining intensity. Transfected cells were identified by dsRED fluorescence. The mean intensity of O-GlcNAc staining in the cell body was measured for each cell using a region of interest outlining the periphery of the cell body. No differences were found in the areas of the cell bodies across measurement groups (data not shown), indicating that they were equivalent and changes in mean intensity measurements were not attributable to changes in cell body spreading. Background was subtracted from each measurement as follows; the mean intensity of a region on the substratum adjacent to each neuron measured was determined using the same region of interest as used for the measurement on the neuron and was subtracted from the measurement obtained from the neuron.

### Morphometrics

Image acquisition was performed on a Zeiss Axiovert 200M microscope (Zeiss, Gottingen, Gewrmany) using a 20× objective and an Orca ER CCD camera (Hamamatsu, Bridgewater, NJ). The microscope and camera are in series with a dedicated PC workstation running Zeiss Axiovision software for image acquisition and quantitative morphometric analysis.

For determination of axon and minor process length, processes were measured using the segmented length measurement module of Axiovision. The measurements reflect the entire length of the process and not the distance from axon tip to the cell body as approximated by a line. Measurements of axon and minor process length were obtained from the microtubule staining pattern. The number of filopodia and lamellipodia were determined by the phalloidin staining pattern revealing actin filaments, the major cytoskeletal elements of filopodial and lamellipodia. The numbers of branches were determined from the combined microtubule and F-actin staining patterns. In order for a protrusion extending from the axon shaft to be considered an axonal branch, it had to be greater than 10 μm in length and contain microtubules. In the transfection experiments, protrusions extending from the axon shaft greater than 10 μm in length were considered axon branches.

### Western Analysis

Neurons were cultured for 8–48 h prior to harvesting for western analysis. A total of 10^6^ neurons were harvested per sample. Neurons were scraped off the substratum, pelleted via centrifugation, and lysed in RIPA buffer as described in Slawson et al. (2005). Cell scraping and pelleting were required in order to obtain protein samples sufficiently concentrated for western analysis. Samples were run on 10% SDS polyacrylamide gels with a 3% stacking gel and transferred to Immobilon-P membranes (Millipore, Billerica, MA). Membranes were blocked with 1% BSA + 0.05% Tween and stained using primary antibody. Following washing, membranes were stained with an appropriate HRP conjugated secondary antibody and developed using ECL Plus Western Blotting Detection System (GE Healthcare, Piscataway, NJ) following the manufacturer's directions. Membranes were imaged using Kodak Biomax XAR film (Fischer, Pittsburg PA; 30-s exposure). Membranes were stripped and reprobed with anti-actin antibodies (Sigma, 2006 monoclonal) to determine loading controls. In some cases, membranes were stained with Ponceau S (Fluka Biochemika) to examine equal protein loading. Primary antibodies used in Western blotting were Anti-O-GlcNAc (110.6) IgM monoclonal (Comer et al., 2001; p. 134), Anti-Phospho-(Ser/Thr) PKA Substrate (Cell Signaling) rabbit polyclonal IgG, mouse monoclonal anti-β actin clone AC-15 (Sigma), and a rabbit polyclonal anti-OGT (kind gift of Gerald Hart, Johns Hopkins). Densitometry of bands on Western blots was performed on the Alpha Innotech— AlphaImager 3400.

### Statistical Analysis

Although some data set are presented as normalized values, the statistical analysis was performed on the absolute data values in the case of *t*-tests and on the number of neurons per category in the case of χ^2^ tests. Analysis was performed using Microsoft Excel or Instat3 (GraphPad Software, La Jolla, CA). Since the nature of the investigations was largely exploratory seeking to determine if altering O-GlcNAc levels has any effect on neuronal morphogenesis, we routinely used 2-tailed *t*-tests. Throughout the manuscript, data are presented as means ± SEM.

## RESULTS

### Distribution of O-GlcNAc-Containing Proteins in Cultured Forebrain Neurons

**Figure 1:**
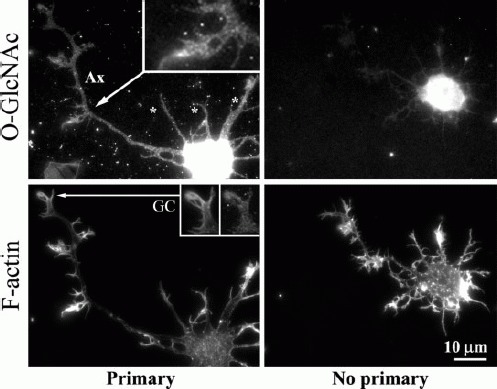
Distribution of O-GlcNAc-modified proteins in cultured forebrain neurons (48 h). Panels on the left show a representative neuron double labeled with anti-O-GlcNAc and phalloidin to reveal actin filaments. The right panels show a neuron stained only with the secondary antibody. Cells were stained in parallel and images acquired using identical settings. Nonspecific staining is evident in the cell body, but not in the processes. O-GlcNAc-modified proteins are found throughout both minor processes (*), the axon (Ax), protrusions from the axon shaft (inset in top left panel), and in the growth cone (GC). The staining pattern is punctate in processes and protrusions from the processes.

We used immunofluorescent staining with an antibody specific to O-GlcNAc to determine the localization of O-GlcNAc-modified proteins in primary cultures of developing forebrain neurons. O-GlcNAc staining was nonuniform, being particularly intense in the cell body, and punctuate in all neuronal processes (see Fig. 1), as well as localizing to axonal filopodia/lamellipodial protrusions and in the growth cone (Fig. 1 insets). No primary antibody controls (see Fig. 1) revealed nonspecific staining in the cell body, but this nonspecific staining was less than that observed with the inclusion of the primary. A low level of nonspecific staining was visible in neuronal processes, possibly due to nonspecific binding of the secondary antibody or intrinsic cell fluorescence, but the intensities of the nonspecific binding were clearly less than those observed with primary antibody (see Fig. 1). O-GlcNAc staining was clearly present in the growth cones of axons and minor processes (see Fig. 1). The distribution of O-GlcNAc-modified proteins in neuronal processes suggests that this post-translational modification may have direct roles in process formation and/or extension.

### Overexpression of O-GlcNAcase Decreases Neuronal O-GlcNAc Levels

To determine cellular roles for O-GlcNAc modifications in neuronal development, we examined the influence of experimentally decreasing levels of O-GlcNAc. Reducing levels of O-GlcNAc in cells by knock-down or knock-out of the transferase that adds O-GlcNAc to protein (OGT) has not been successful, as loss of OGT is lethal to cells (Shafi et al., 2000; O'Donnell et al., 2004). Therefore, we sought to genetically lower O-GlcNAc levels by overexpressing O-GlcNAcase, the enzyme that removes O-GlcNAc from proteins. O-GlcNAcase was cotransfected with dsRED and control cells were transfected with dsRED alone. Cotransfection with equal amounts of dsRED and eGFP resulted in 88% of neurons expressing both dsRED and eGFP (*n* = 176), demonstrating that cotransfection is a reliable approach in our system. Furthermore, in the experimental cotransfection 1.3-fold more O-GlcNAcase plasmid was used than the dsRED reporter in order to maximize the numbers of dsRED expressing neurons that would also coexpress O-GlcNAcase. O-GlcNAc levels and the morphology of neurons were analyzed 48 h after transfection.

**Figure 2:**
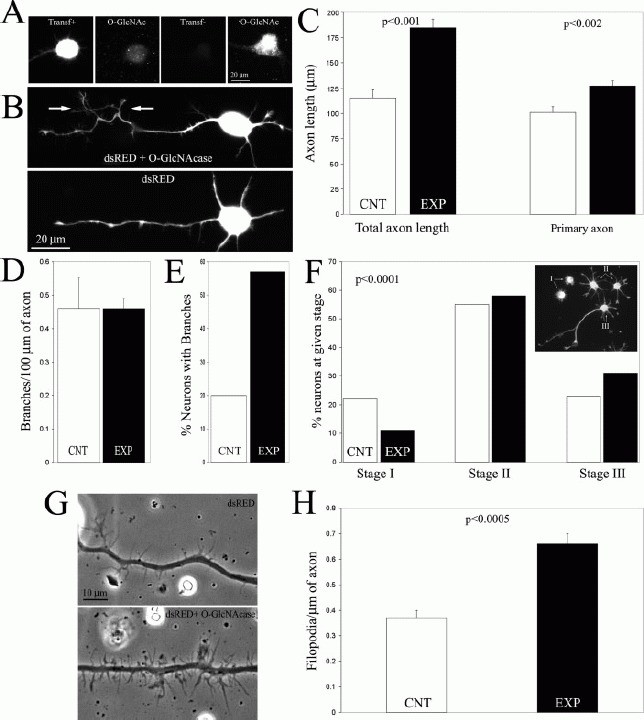
Over-expression of O-GlcNAcase promotes axon branching. (A) Example of O-GlcNAc staining in a neuron transfected with O-GlcNAcase (Transf+, dsRed channel) and not transfected (Transf−) from the same culture. Images were acquired identically. The Transf− neuron contain normal levels of O-GlcNAc staining while the Transf+ neuron exhibits minimal staining, which is likely background due to the spherical soma of the neuron. DsRed expression alone did not alter O-GlcNAc staining (not shown). (B) Examples of transfected neurons. The bottom panel shows the characteristic morphology of a dsRED alone transfected neuron. The top panel shows a dsRED + O-GlcNAcase transfected neuron. In both cases the dsRED signal is shown. Arrows point to axonal branches. (C) Quantification of total (primary axon + axon branches) axon length and the length of the primary axon. *n* = 60 and 140 neurons for the dsRED (control, CNT) and dsRED + O-GlcNAcase (experimental, EXP) groups, respectively. (D) Quantification of the mean number of primary axon branches per axon length considering only axons with branches. *n* = 12 and 81 for CNT and EXP groups, respectively. (E) Percentage of neurons exhibiting one or more axon branches as a function of dsRED expression (CNT) or dsRED + O-GlcNAcase (EXP). (F) Characterization of developmental stages exhibited by transfected neurons. χ*^2^* test. *N* = 260 and 595 for CNT and EXP groups, respectively. (G) Phase contrast examples of transfected axons (dsRED channel not shown). (H) Quantification of the mean number of filopodia per unit length of axon. *n* = 44 and 59 for CNT and EXP groups, respectively. All data compared using 2-tailed Welch *t*-tests unless otherwise noted.

Since we obtain a transfection efficiency of ∼20–30%, determination of the effects of O-GlcNAcase expression on O-GlcNAc levels is not feasible using Western blotting approaches. Therefore, we determined the effects of O-GlcNAcase expression on O-GlcNAc levels at the single cell level using immunocytochemistry. Expression of O-GlcNAcase greatly reduced O-GlcNAc levels in neurons [Fig. 2(A)]. To determine the relative decrease in O-GlcNAc levels resulting from overexpression of O-GlcNAcase, we measured the intensity of staining in neurons expressing dsRED, dsRED + O-GlcNAcase, and nontransfected neurons in culture transfected with dsRED alone or dsRED + O-GlcNAcase. For this analysis, we measured the staining intensity in Stage II neuronal cell bodies at 48 h of culturing. Comparison of the staining intensity in neurons expressing dsRED alone (*n* = 23) to that in nontransfected neurons from cultures transfected with either dsRED (*n* = 23) or dsRED + O-GlcNAcase (*n* = 25) did not reveal a difference (*p* > 0.32 and *p* > 0.21 respectively, 2-tailed Welch *t*-test), indicating that dsRED transfection alone did not alter O-GlcNAc levels. Similarly, there was no difference between nontransfected neurons in the dsRED and dsRED + O-GlcNAcase groups (*p* > 0.25, 2-tailed Welch *t*-test), demonstrating that the baselines across groups were comparable. However, comparison of the staining intensity in neurons transfected with dsRED + O-GlcNAcase (*n* = 23) to dsRED transfected neurons revealed a 61% decrease in O-GlcNAc staining intensity (*p* < 0.00001, 2-tailed Welch *t*-test). Thus, overexpression of O-GlcNAcase is an effective method for decreasing neuronal O-GlcNAc levels.

### Overexpression of O-GlcNAcase Increases the Percentage of Neurons Exhibiting Axon Branching Without Affecting the Frequency of Branches Per Unit Length of Axon

O-GlcNAcase transfected neurons exhibited more complex axonal morphologies relative to controls expressing dsRED alone [Fig. 2(B)]. Expression of O-GlcNAcase resulted in an increase in the percentages of neurons with axons that exhibited branching. Indeed, 57% (*n* = 140) of O-GlcNAcase over-expressing neurons exhibited one or more branches relative to 20% (*n* = 60) of dsRED alone expressing control neurons [Fig. 2(E)]. Thus, decreases in O-GlcNAc levels induced by overexpression of O-GlcNAcase resulted in a 1.85-fold increase in the percentage of neurons that exhibited axon branching.

O-GlcNAcase over-expression resulted in a 50% increase in the mean total axon length (primary axon + axon branches) per neuron [Fig. 2(B,C)]. However, since the length of the primary axon alone increased by only 26% [Fig. 2(C)], the increase in total axon length was partially due to increased branching of axons [Fig. 2(B)]. If all neurons are considered regardless of whether their axons had branches or not, analysis of the frequency of axon branching per unit length of axon across populations reveals a 180% increase in O-GlcNAc over-expressing neurons relative to controls (*p* < 0.001, 2-tailed Welch *t*-test). However, the frequency of branches per unit length of the primary axon did not differ between O-GlcNA-case over-expressing neurons and dsRED expressing controls when only neurons with branches were considered [Fig. 2(D)]. Therefore, these data indicate that reduction of O-GlcNAc levels by O-GlcNAcase over-expression induces a branching phenotype in neurons, but does not regulate the frequency at which branches are formed from the axons. These data indicate that endogenous levels of O-GlcNAc act as negative regulators of the probability that a given neuron will exhibit axon branching, but once the branching program is activated neurons form the same numbers of branches per unit length of axon as observed in control neurons that generate branches.

Cultured forebrain neurons undergo a characteristic sequence of development resulting in the formation of a single neuronal axon and multiple minor processes, which are dendritic precursors (Heidemann et al., 2003). The generation of a single axon from pre-existing minor processes is referred to as the acquisition of neuronal polarity (reviewed in Arimura and Kaibuchi, 2007). The sequence of neuronal development *in vitro* proceeds through three stages [Fig. 2(F) inset]. Following attachment to the substratum, the neurons enter Stage I and elaborate filopodial and lamellipodial protrusions from their cell bodies. Stage II is characterized by the emergence of multiple short processes termed minor processes. Finally, neuronal polarity is attained at Stage III when one of the minor processes begins rapid extension and gives rise to a single axon. Over-expression of O-GlcNAcase resulted in a shift toward more Stage III, and less Stage I, neurons [Fig. 2(F)], suggesting that decreasing O-GlcNAc levels allows neurons to more rapidly enter and exit Stage II of development. However, the overall change in the distribution of neurons at various stages in response to decreased level of O-GlcNAc was relatively minor, and may reflect nonspecific differences induced by lowered levels of O-GlcNAc such as minor alterations in vesicular traffic or rates of cell attachment.

Axonal filopodia are precursors to axon branches (Gallo and Letourneau, 1999). Although analyzing the overall morphology of neurons, we observed that Stage III O-GlcNAcase transfected axons appeared to have greater numbers of filopodia [Fig. 2(G)]. Indeed, O-GlcNAcase over-expressing neurons exhibited 78% more filopodia per unit length of axon [Fig. 2(H)]. In summary, O-GlcNAcase over-expression increased the number of axonal filopodia and the percentage of neurons that generated axon branches.

### Increasing Levels of O-GlcNAc-Modified Proteins Does Not Affect Neuronal Process Extension

**Figure 3:**
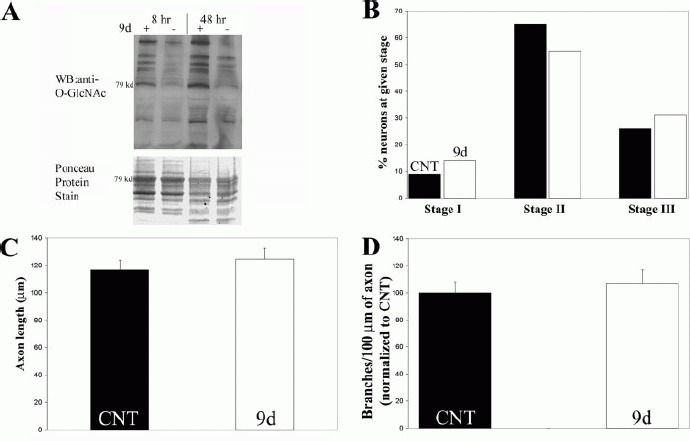
Inhibition of O-GlcNAcase using 200 μ*M* 9d does not alter the development of neuronal polarity. (A) Western blot (WB) analysis of O-GlcNAc modifications in cultured forebrain neurons either untreated or 9d treated for 8 and 48 h of *in vitro* development. Treatment with 9d greatly increased the levels of O-GlcNAc-modified proteins as early as 8 h. Ponceau protein stain was used to determine relative loading across samples. (B) 9d did not affect the development of neuronal polarity *(χ^2^* test, *p* > 0.3). The experiment was performed in quadruplicate with ≥100 neurons scored per experiment. (C) 9d did not alter the total length of axons (primary axon + axon branches; 2-tailed Welch t-test). *n* = 90–94 axons per group. (D) 9d did not change the branching frequency of axons (2-tailed Welch *t*-test). *n* = 155–173 axons per group. All data obtained from 48 h old cultures.

9d is a selective inhibitor of O-GlcNAcase, and has been used to specifically increase levels of O-GlcNAc modifications on proteins in cell culture (Macauley et al., 2005). We first verified that 9d increased the levels of O-GlcNAc modifications on proteins in our culturing system using Western blot analysis. Forebrain cultures were treated for 8 or 48 h with 9d starting at the time of plating, and equal levels of protein from cell lysates were analyzed by Western blotting using an O-GlcNAc specific antibody. We observed multiple O-GlcNAc positive bands in control neurons at both 8 and 48 h [Fig. 3(A)]. Although the overall levels of O-GlcNAc reactivity at 8 and 48 h of control cells did not change appreciably, the intensity of O-GlcNAc reactivity in several specific bands was altered between 8 and 48 h, suggesting potential dynamic O-GlcNAc modification of some proteins between 8 and 48 h *in vitro* development [Fig. 3(A)]. Treatment with 9d elevated O-GlcNAc levels in the majority of protein bands at 8 h, and this elevation persisted through 48 h [see Fig. 3(A)]. The importance in demonstrating maximal O-GlcNAc elevation at 8 h is that by this time point in culture, neurons have attached to the substratum but not yet elaborated processes. Thus, 9d increased O-GlcNAc levels in neurons during the time period when the neurons extend processes.

We first sought to determine if increasing O-GlcNAc levels using 9d altered the progression through Stages I, II, and III of neuronal development. 9d treatment of cultures from the time of plating did not affect the progression of cultured forebrain neurons through Stages I–III [Fig. 3(B)]. We next determined whether 9d alters the length of axons. Stage III cells were selected for analysis as only cells at this stage exhibit axons. Measurements of axon length did not reveal an effect of 9d on mean axon length [Fig. 3(C)] or branching [Fig. 3(D)].

### Increasing Levels of O-GlcNAc Modifications Decreases Axonal Filopodial Numbers

**Figure 4:**
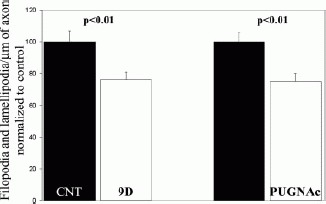
Inhibition of O-GlcNAcase with either 9d or PUGNAc decreases the numbers of axonal filopodia and lamellipodia. Cultures were treated with 200 μM 9d or 100 μM PUGNAc at the time of plating. Both 9d and PUGNAc treatments increased O-GlcNAc levels to a similar extent (data not shown) at 48 h after treatment, and decreased the number of filopodia and lamellipodia along axons compared to control vehicle treatment (Cnt). 2-tailed Welch *t*-test. *n* = 81–94 axons per group. Baseline filopodial numbers were ∼0.37/μm of axon (see Figure 2H). All data obtained from 48 h old cultures.

Although treatment with 9d did not affect the overall development of cultured forebrain neurons, we observed a simplification of axonal morphology reflected in decreased numbers of axonal filopodial and lamellipodial protrusions (see Fig. 4). Measurement of the number of filopodia/lamellipodia per unit length of axons revealed that 9d decreased the number of protrusions along axons by 25% (see Fig. 4). To confirm this observation, we next cultured neurons in the presence of a different O-GlcNAcase inhibitor known as PUGNAc (Haltiwanger et al., 1998). Western analysis of O-GlcNAc levels in the presence of PUGNAc revealed effects similar to those of 9d (data not shown). PUGNAc treatment decreased the number of axonal protrusions to a similar extent as 9d (see Fig. 4). Collectively, these data indicate that the degree of O-GlcNAc modification of neuronal proteins regulates the number of axonal protrusions.

### Increasing Levels of O-GlcNAc Modifications Blocks Forskolin-Induced Axon Branch Formation

**Figure 5:**
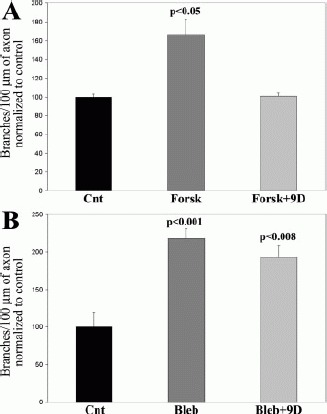
Inhibition of O-GlcNAcase with 9d blocks for-skolin-induced increases in axon branching. (A) Cultures were treated with 50 μ*M* forskolin +/− 200 μ*M* 9d at the time of plating. Control (Cnt) cultures were treated with vehicle for the drugs. Forskolin (Forsk) increased the number of branches per unit axon length at 48 h. Cotreatment with 9d and Forsk prevented the increase in branches induced by forskolin. Comparison of Cnt with Forsk +9d did not reveal a difference (2-tailed Welch *t*-test). 9d treatment alone did not affect the number of branches relative to Cnt (Figure 3D). *n* = 70–83 axons per group. (B) Cultures were treated with 50 μ*M* blebbistatin +/− 200 μ*M* 9d at the time of plating. Control cultures were treated with vehicle for the drugs. Blebbistatin (Bleb) increased the number of branches at 48 h. However, 9d did not block the increase in branch number induced by Bleb. *n* = 84–103 axons per group.

Cyclic-AMP (cAMP) signaling is an important regulator of axon growth and branching (Weeks et al., 1991; reviewed in Cui and So, 2004). Griffith and Schmitz (1999) demonstrated a reciprocal relationship between the cAMP-protein kinase A signaling axis and O-GlcNAc levels on cytoskeletal proteins of cultured primary neurons. Given that reducing O-GlcNAc levels caused increased axon branching, we sought to determine if axon branching driven by activation of the cAMP axis would be sensitive to the levels of O-GlcNAc modifications. Culturing fore-brain neurons for 48 h in the presence of forskolin (50 μM), an activator of adenylyl cyclase resulting in increased cAMP production (Seamon and Daly, 1981) increased the number of axon branches [Fig. 5(A)], without affecting axonal filopodial numbers (data not shown). Cotreatment of cultures with forskolin and 9d blocked the increase in axon branches induced by forskolin treatment [Fig. 5(A)].

We next tested whether 9d treatment would alter increases in axon branch number induced by inhibition of the mechanoenzyme myosin II (Rosner et al., 2007). Myosin II was inhibited using 50 μM blebbistatin (Straight et al., 2003). Culturing neurons in the presence of blebbistatin significantly increased the number of axon branches [Fig. 5(B)]. However, 9d did not affect the number of branches elicited by blebbistatin treatment [Fig. 5(B)]. Thus, 9d blocks increases in axon branches induced by forskolin but not by inhibition of myosin II.

### Increasing Levels of O-GlcNAc Modifications Blocks Specific Forskolin-Induced Phosphorylation Events

**Figure 6:**
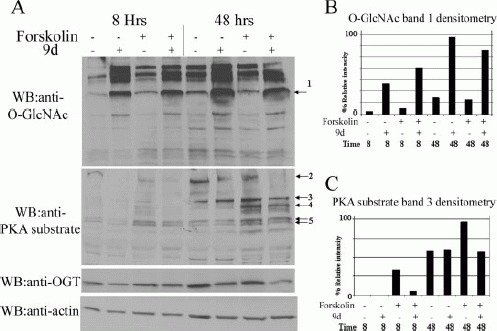
Inhibition of O-GlcNAcase with 9D blocks forskolin-induced increases in PKA substrate phosphorylation. (A) Cultures were treated with 50 μ*M* forskolin +/− 200 μ*M* 9D at the time of plating. Control (-) cultures were treated with vehicle for the drugs. At 8 and 48 h, cultures were lysed for Western blotting to examine levels of O-GlcNAc, PKA substrate phosphorylation, OGT, and actin. Forskolin (Forsk) induced several PKA substrate phosphorylated events (labeled as bands 2, 3, 4, and 5) that were inhibited by 9d elevation of O-GlcNAc levels. Densitometry to quantify relative levels of an O-GlcNAc band (labeled as band 1) (B) and a PKA substrate phosphorylated band (labeled as band 3) (C) was performed. Forskolin-induced phosphorylation of the PKA substrate band 3 was reduced by 84% and 41% by 9d elevation of O-GlcNAc at the 8- and 48-h time points, respectively. The results are representative of three individual experiments.

PKA activation likely contributes to forskolin-induced axon branching. Given the precedent of interplay between serine/threonine phosphorylation and O-GlcNAc modifications, it was possible that elevated O-GlcNAc interference with specific PKA phosphorylation signaling events contributed mechanistically to 9d inhibition of forskolin-induced axon branching. Thus, we examined the effects of elevated O-GlcNAc on phosphorylation of PKA substrates. Neurons were cultured for 8 or 48 h, and O-GlcNAc modifications and PKA substrate phosphorylation were examined by Western blotting in response to treatment with forskolin, 9d, or a combination of these [Fig. 6(A)]. At 8 and 48 h, several forskolin-induced PKA substrate phosphorylation events [labeled as bands 2, 3, 4, and 5 at right of blot in Fig. 6(A)] are reduced by 9d elevation of O-GlcNAc [Fig. 6(A)]. Using densitometry, the relative intensity of a representative PKA substrate was determined [Fig. 6(C), band 3 in panel A]. Densitometry was also used to quantify elevation of O-GlcNAc on a selected band [Fig. 6(B), band 1 in panel A]. In all cases, 9d caused a strong elevation in O-GlcNAc reactivity with this band. Furthermore, consistent with Figure 3(A), at 48 h there is an increase in O-GlcNAc levels compared to the 8-h time point in the absence of any treatments [Fig. 6(A,B)]. This indicates that upregulation of OGT expression and the extent of O-GlcNAc modification of proteins occur across neuronal development *in vitro* and may be a part of the normal program of neuronal morphogenesis. Western blotting to detect actin confirms equal protein loading at either the 8- or 48-h time points. Thus, it appears that 9d elevation of O-GlcNAc inhibits axon branching at least in part through attenuating PKA signaling.

## DISCUSSION

Post-translational modifications of proteins are a fundamental mechanism for the regulation of cellular function. Although O-GlcNAc modification of proteins was discovered over two decades ago (Torres and Hart, 1984), little is known about the functions of O-GlcNAc in neuronal cell biology, and roles for O-GlcNAc in primary neurons have not been examined. Our data indicate that O-GlcNAc protein modifications have a role in the overall development of cultured embryonic forebrain neurons during early stages of their morphologic differentiation, resulting in neuronal polarization. Moreover, O-GlcNAc protein modifications are involved in regulating the numbers of axonal filopodia and the ability of axons to elaborate branches.

The sprouting of axon branches is a common response to injury, and it is also of fundamental importance to the establishment of connectivity patterns in the developing nervous system (Deller et al., 2006). Over-expression of O-GlcNAcase promoted the formation of axon branches. However, over-expression of O-GlcNAcase did not increase the number of axon branches per unit length of the axon, rather it increased the proportion of neurons that exhibited axon branches. Thus, decreased levels of O-GlcNAc modifications allow neurons to enter a branching program and elaborate axon branches. However, pharmacologically increasing the levels of O-GlcNAc on neuronal proteins did not decrease axon branching. This discrepancy suggest that endogenous levels of O-GlcNAc are sufficient to maintain neurons in a low-sprouting mode, and that increasing those levels do not cause further inhibitory effects on branching. Alternatively, 20% of neurons that normally exhibit axon branching phenotypes may be operating using a branching mechanism independent of O-GlcNAc regulation and thus unresponsive to increases in O-GlcNAc levels. The downregulation of O-GlcNAc levels in neurons may be a useful approach for promoting the morphologic plasticity of axons in the context of injury and regeneration.

The staining pattern of O-GlcNAc-modified proteins in the cytoplasm of neurons is punctate. Although the significance of this observation is not clear, it may reflect the presence of O-GlcNAc-modified proteins on vesicles. Alternatively, O-GlcNAc-modified proteins may also be found in particles of proteins undergoing slow axonal transport in axons (Roy et al., 2007) or complexes of proteins. The presence of O-GlcNAc-modified proteins in axonal filopodial and lamellipodia is consistent with the data demonstrating that O-GlcNAc modifications regulate axonal filopodia and branch number. It is possible that modulation of O-GlcNAc levels may alter the development of neuronal morphology at later stages of development than examined in our study, when dendrites differentiate from the minor processes and synaptogenesis begins. Indeed, many synaptosomal proteins are O-GlcNAc modified (Vosseller et al., 2006) and axonal filopodia are precursors to synapse formation (reviewed in Jontes and Smith, 2000). Since modulation of O-GlcNAc levels alters the number of axonal filopodia, it is possible that O-GlcNAc plays developmental roles in regulating formation of synapses. These issues will be addressed in future work.

It is not known which specific protein targets of O-GlcNAc modification events are functionally linked to the mechanism of axonal filopodia and branch formation, but O-GlcNAc regulation of these cellular events may be complex, as many neuronal proteins are modified by O-GlcNAc (reviewed in Lefebvre et al., 2005). Moreover, cytoskeletal/microtubule dynamics and membrane vesicle traffic are major determinants of filopodia and axonal branches (Kalil et al., 2000), and proteins that function in these processes are known to be heavily modified by O-GlcNAc. For example, proteins involved in microtubule dynamics that are modified by O-GlcNAc include microtubule-associated proteins 1, 2, and 4 (Ding and Vandré, 1996), tubulin polymerization promoting protein (Vosseller et al., 2006), and Tau (Liu et al., 2004).

The determination that inhibition of O-GlcNacase prevented the increase in axonal branches induced by forskolin suggests regulatory involvement of O-GlcNac modification on proteins involved in cAMP signaling. Our results provide a functional correlate to a previous study demonstrating a reciprocal regulation between phosphorylation and O-GlcNAc on neuronal cytoskeletal proteins downstream of cAMP-PKA signaling (Griffith and Schmitz, 1999). The absence of an effect of inhibition of O-GlcNacase on branches induced by inhibition of myosin II, a molecule that directly regulates branch formation through its interactions with actin filaments, indicates that O-GlcNac modifications are not involved in the regulation of myosin II and that the basic mechanisms underlying branch formation are not affected by 9d treatment. Consistent with this notion, 9d treatment alone did not affect basal numbers of axon branches. Western analysis revealed that the effect of increasing cAMP levels, using forskolin, on the phosphorylation of PKA substrates was antagonized by increasing O-GlcNAc levels, using 9d. These data suggest that cAMP-induced changes in phosphorylation may underlie the effects of cAMP on axon branching (Weeks et al., 1991), and show a correlation between the block of cAMP-induced axon branching and the prevention of forskolin-induced changes in protein phosphorylation in response to increased levels of O-GlcNAc. Collectively, the data indicate that O-GlcNAc modifications are involved in modulating cAMP signaling that in turn promotes branch formation. Although consideration of additional pathways that may be involved in the regulation of axon branching by O-GlcNAc is speculative, GSK and Rac1 are two potential targets in the O-GlcNAc regulatory system that may also be involved in the regulation of axon branching (Ding and Vandré, 1996; Kuhn et al., 2000; Cole and Hart, 2001; Kneass and Marchase, 2005; Kim et al., 2006; Wang et al., 2007).

O-GlcNAc is abundant in the nucleus and has been found on many transcription factors. Thus, influences at the transcriptional level may also contribute to effects of modulating O-GlcNAc. The time course of effects examined in this study in response to altered O-GlcNAc was long enough to involve effects at the transcriptional level on axonal morphology. Clearly, the mechanisms by which O-GlcNAc modifications regulate neuronal development will require further analysis.

In conclusion, we present data that O-GlcNAc protein modifications are involved in specific aspects of the morphological development of cultured embryonic forebrain neurons. We conclude that O-GlcNAc protein modifications are involved in the regulation of axonal filopodia and branching, and also regulate cAMP-induced axon branches. Future work will have to address the mechanisms through which O-GlcNAc modification of specific proteins underlies influences on filopodia and branch formation.
